# Limb Injuries and Disability in the Southwest Region of Cameroon

**DOI:** 10.5435/JAAOSGlobal-D-22-00148

**Published:** 2023-02-16

**Authors:** Fonje Mouansie Ahmed Nour, Madeline S. Tiee, Rasheedat A. Oke, Girish N. Motwani, Kareen E. Azemafac, Susana N. Mbeboh, Frida N. Embolo, Drusia C. Dickson, Rochelle A. Dicker, Catherine Juillard, S. Ariane Christie, Alain Chichom-Mefire

**Affiliations:** From the Faculty of Health Sciences, University of Buea, Buea, Cameroon (Dr. Ahmed Nour, Dr. Azemafac, Dr. Mbeboh, Dr. Embolo, and Dr. Chichom-Mefire); the Center for Global Surgical Studies, Department of Surgery, University of California, San Francisco, San Francisco, CA (Dr. Ahmed Nour, Dr. Tiee, Motwani, Dr. Azemafac, Dr. Mbeboh, Dr. Embolo, Dr. Dickson, and Dr. Christie); the Department of Orthopaedic Surgery and Rehabilitation, Loyola University Medical Center, Maywood, IL (Tiee); and the Program for the Advancement of Surgical Equity, Department of Surgery, University of California, Los Angeles, Los Angeles, CA (Dr. Oke, Dr. Dicker, and Dr. Juillard).

## Abstract

Extremity injuries are a leading cause of morbidity in low- and middle-income countries (LMICs), often resulting in marked short-term and long-term disabilities. Most of the existing knowledge on these injuries originates from hospital-based studies; however, poor access to health care in LMICs limits these data because of inherent selection bias. This subanalysis of a larger population-level cross-sectional study in the Southwest Region of Cameroon aims to determine patterns of limb injury, treatment-seeking behaviors, and predictors of disability.

**Methods:**

Households were surveyed in 2017 on injuries and subsequent disability sustained over the previous 12 months using a three-stage cluster sampling framework. Subgroups were compared using the chi square, Fisher exact, analysis of variance, Wald, and Wilcoxon rank-sum tests. Logarithmic models were used to identify predictors of disability.

**Results:**

Of 8,065 subjects, 335 persons (4.2%) sustained 363 isolated limb injuries. Over half of the isolated limb injuries (55.7%) were open wounds while 9.6% were fractures. Isolated limb injuries most commonly occurred in younger men and resulted from falls (24.3%) and road traffic injuries (23.5%). High rates of disability were reported, with 39% reporting difficulty with activities of daily living. Compared with individuals with other types of limb injuries, those with fractures were six times more likely to seek a traditional healer first for care (40% versus 6.7%), 5.3 times (95% CI, 1.21 to 23.42) more likely to have any level of disability after adjustment for injury mechanism, and 2.3 times more likely to have difficulty paying for food or rent (54.8% versus 23.7%).

**Discussion:**

Most traumatic injuries sustained in LMICs involve limb injuries and often result in high levels of disability that affect individuals during their most productive years. Improved access to care and injury control measures, such as road safety training and improvements to transportation and trauma response infrastructure, are needed to reduce these injuries.

Injury is a leading cause of morbidity and mortality worldwide with more than 5 million deaths annually, of which an estimated 90% occur in low- and middle-income countries (LMICs).^1^ Unfortunately, recent increases in motor vehicle volume have not been accompanied by commensurate improvements in road safety infrastructure, and trauma deaths are expected to rise.^1^ Limb injuries make up an estimated 49% to 67% of all traumatic injuries in sub-Saharan Africa and cause considerable morbidity.^2-6^ Potential complications of limb injuries include amputation, soft-tissue infection, osteomyelitis, malunion, nonunion, and posttraumatic arthritis and may lead to chronic pain, functional impairment and disability, and psychosocial effects such as depression or posttraumatic stress disorder.^4,7-12^ A population-based survey in Ghana found that 78% of patients with long-term disability after injury sustained trauma to the extremities.^7^ Furthermore, most of the injured patients seen in regional hospitals are men between 20 and 49 years, a population in which disability can result in particularly serious socioeconomic consequences for patients and their families.^7^ Despite this, relatively little data exist characterizing the relationship between patterns of limb injury, treatment type, and disability.

Most existing studies on limb injuries rely on hospital-based registries and fail to include data on injured persons who do not present to formal health care. Relatively few studies have been conducted using population-level surveys to understand limb-injury incidence and patterns in various communities.^4,6^ In a study conducted in Ghana, approximately one-third of participants with an extremity injury that caused more than 1 month of disability did not seek any formal care.^6^ Another study conducted in Cameroon revealed that approximately 31% of school children who were injured did not receive formal care.^13^ Owing to the high number of patients who do not present to formal care, more robust population-level studies are needed to understand patterns of injury, outcomes of injury, and factors related to disability.

In 2017, we conducted a regional population-level mixed-methods survey to characterize the burden of injury in Cameroon's Southwest Region (SWR).^14^ To better understand the contribution of limb injuries to disability and outcomes in sub-Saharan Africa, we report here the findings of a subanalysis of these population-level data. We hypothesize that, like in other countries in sub-Saharan Africa, limb injuries would account for a large portion of Cameroon's burden of injury and contribute markedly to overall disability. In addition, we predict that individuals who do not seek formal care or seek other treatment options first will experience higher rates and severity of disability.

## Methods

### Study Design, Sampling Strategy, Study Population, and Survey Administration

We conducted a subanalysis of a larger community-based cross-sectional study designed to identify the incidence of injury in the SWR of Cameroon between January and March 2017. The SWR has a mixed urban-rural distribution and consists of 18 health districts with a combined population of 1,575,224.^15^ Sampling was done using a three-stage cluster sampling framework. Details of the sampling strategy and sample size calculation have been previously described.^14^ Briefly, sampling was done first at the health district level and then at the level of health areas using probability of selection proportionate to population size. The final stage involved the selection of households (200 per health area). A minimum sample size of 4,680 individuals was calculated for the overall study, which was deliberately exceeded during collection by 50% at each site to allow for multiple subanalyses of rare events.^14^

We excluded households in which members had not lived in the SWR for at least 6 months in the past year, households in which no member present could consent (ie, no member present was older than 18 years), and households where no one was home after two attempts. Using a standard script, oral consent was obtained from the household representative by trained Cameroonian research assistants who then administered a pretested questionnaire to the representative regarding all members of the household. Information collected included demographics of the household and each household member and whether they had sustained any limb injury. For each limb injury event, information about the location and activity at the moment of injury, the mechanism of injury, the specific limb injured and type of injury, the number of school or work days missed, care-seeking behavior, cost of treatment, disability, and economic consequences was collected. Disability was measured using standard disability categories adapted from the World Health Organization's (WHO) Guidelines for Conducting Community Surveys on Injuries and Violence.^16^ Disability was self-reported as mild, moderate, or severe for various daily activities and mental health including any new or worsened difficulty with (1) dressing, eating, or going to the restroom; (2) leaving home, shopping, or traveling; (3) going to school; (4) standing or walking; (5) picking things up or using their hands; and (6) depression or shame. Individuals were able to report disability in multiple categories. As outlined in the WHO guidelines, individuals with mild disability were still independent in their activities; those with moderate disability required some help in performing activities; and those with severe disability were unable to perform activities.^16^

Ethical approval was obtained from the University of Douala Ethical Review Board and the University of California, San Francisco's Institutional Review Board, and administrative approval was obtained from the regional delegate of public health of the SWR. All individuals involved in data collection, management, and analysis received an online course on Human Subjects Research from the Collaborative Institutional Training Initiative Program.

### Data Management and Analysis

All data were entered at regular intervals into REDCap,^17,18^ a secure online database application hosted on the University of California, San Francisco server. Data analysis was conducted using Stata Version 14 and was adjusted for clustering methodology.^19^ Individuals with limb injuries were characterized using means and standard deviations for parametric continuous variables, medians and interquartile ranges for nonparametric continuous variables, proportions for binary variables, and percentages or frequencies for categorical variables. Differences between groups were compared using the chi square, Fisher exact, analysis of variance, Wald, and Wilcoxon rank-sum tests as appropriate. A *P*-value of less than 0.05 was considered statistically significant.

To identify predictors of disability, univariate logarithmic models were used to compare disability groups: (1) any disability versus no disability and (2) severe/moderate disability versus mild/no disability. Predictors used included type of injury, mechanism of injury, activity during injury, first responder on the scene, treatment type first sought, comparisons between various treatment categories, cost of treatment, age, sex, education level, whether a person owned a cellphone, or living in an urban versus rural location. Variables with a strong association with disability were used in a multivariate logarithmic model to allow for interactions between variables. The variables were dropped in a stepwise fashion, and nested models were compared using adjusted Wald tests. Variables with a *P*-value of < 0.05 were considered significant.

## Results

Among the 8,065 subjects surveyed, 471 persons (5.8%) were injured over the past 12 months, sustaining a total of 503 injuries, of which 400 (79.5%) were limb injuries.^14^ Of these 471 people, 380 (80.7%) sustained a limb injury and 335 (71.1%) had isolated limb injuries. Most were isolated limb injuries (N = 363, 90.8%), whereas the other 9.2% (N = 37) occurred in conjunction with injuries to other parts of the body. Of the 400 limb injuries, 31.2% (N = 125) were upper extremity injuries while 68.8% (N = 275) were lower extremity injuries.

### Social and Demographic Factors of Subjects With Isolated Limb Injuries

In comparison with the general survey population, subjects with isolated limb injuries were older with a median age of 24 years (interquartile range [IQR] 14 to 37) compared with 20 years (IQR, 10 to 34) for the survey population. While male patients comprised 46% of the survey population (N = 3,551; 95% CI, 44.0 to 48.5), they sustained 57% of reported limb injuries (N = 191; 95% CI, 46.3 to 67.5) (*P* = 0.041). No other notable demographic differences were found between persons with limb injuries and the overall survey population. Individuals with isolated limb injuries were most commonly students (N = 120, 36.5%) and farm workers (N = 91, 27.7%).

### Patterns in Mechanism of Injury and Care Sought for Isolated Limb Injuries

#### Mechanism and Type of Injury

**Figure 1:**
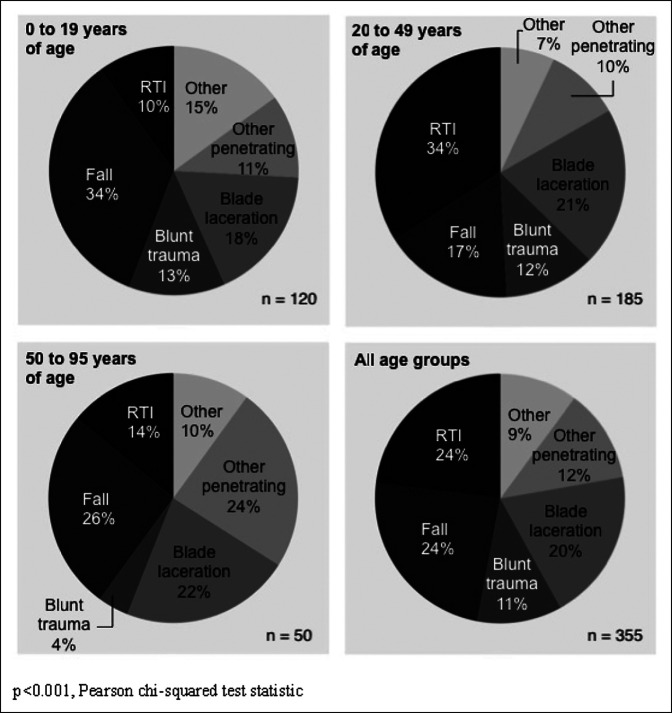
Mechanism of injury by age group for individuals reporting isolated limb injury p<0.001, Pearson chi‐squared test statistic.

The leading mechanisms causing isolated limb injuries were falls (N = 86, 24.3%), followed by road traffic injuries (RTIs; N = 83, 23.5%) and blade lacerations (N = 71, 20.1%) (Figure 1). Notably, the overall distribution of mechanisms leading to isolated limb injury differed significantly (*P* < 0.001) from the mechanisms leading to multiple injuries. Polytrauma most commonly resulted from road traffic injuries, falls, or blunt trauma, whereas isolated limb injuries were more frequently attributable to penetrating trauma (Supplemental Table 1, http://links.lww.com/JG9/A258). Mechanisms also differed by age groups (*P* < 0.001), with RTI being the most common for adults between 20 and 49 years and falls most common in children and adolescents (Figure 1). Moto-taxis were the predominant vehicle involved in RTI leading to limb injuries (N = 47, 56.3%). Isolated limb injuries most commonly consisted of wounds (N = 208, 57.8%), sprains (N = 51, 14.2%), and fractures (N = 35, 9.7%) (Table 1).

**Table 1:** Type of Injury for Subjects With Isolated Limb Injuries Reporting Any Disability Compared With Individuals Who Did Not Report Disability

Type of injury	Total^a^ (N = 363)	Disabled^b^ (N = 256)	Not Disabled^b^ (N = 78)	*P*
	Freq (%)	Freq (%)	Freq (%)	
Wound	208 (57.8)	140 (55.1)	49 (63.6)	0.0003^c^
Sprain	51 (14.2)	48 (18.9)	5 (6.5)	
Fracture	35 (9.7)	31 (12.2)	2 (2.6)	
Dislocation	29 (8.1)	17 (6.7)	0 (0.0)	
Bruise	19 (5.3)	7 (2.8)	9 (11.7)	
Burn	16 (4.4)	10 (3.9)	12 (15.6)	
Pain	1 (0.3)	1 (0.4)	0 (0.0)	

Freq = frequency, %: percentage

a Included all isolated limb injuries reported (one person could report more than one type of limb injury).

b Injury that was most severe of injuries sustained.

c *P*-value less than or equal to 0.05 considered statistically significant; data are survey-adjusted; the Pearson chi square test was used to compare the patients with any disability with those reporting no disability.

#### Patterns in Care Sought

**Figure 2:**
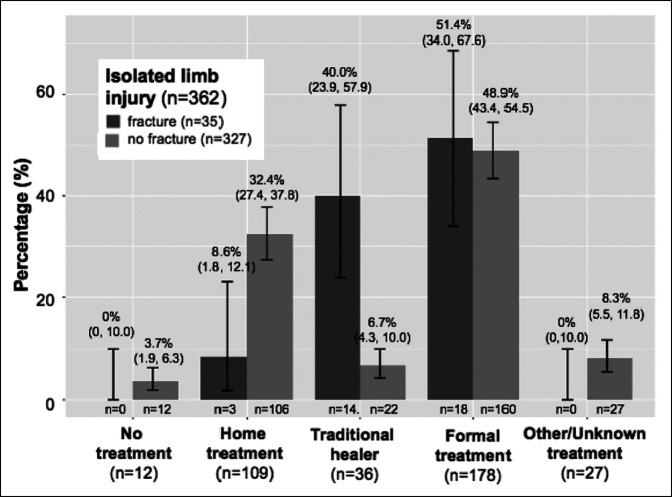
Comparison of treatment first sought for individuals with and without fractures among subjects with isolated limb injuries (95% confidence intervals reported).

Decisions regarding care differed for individuals who sustained isolated limb injuries compared with individuals with limb injuries in conjunction with other injuries (*P* = 0.006). Formal medical care was sought initially by 49.2% (N = 178) of individuals sustaining isolated limb injuries (Figure 2, Supplemental Table 1, http://links.lww.com/JG9/A258). Overall, 60.8% (N = 221) of all subjects with isolated limb injuries sought formal medical care at some point after injury, of which 18.7% (N = 41) sought formal care after first seeking some alternative treatment modality. Individuals with limb fractures were more likely to seek a traditional healer first after injury (40.0%; 95% CI, 23.9 to 57.9 versus 6.7%; 95% CI, 4.3 to 10.0) and more likely to consult a traditional healer at some point during their care (65.7% versus 9.2%, *P* < 0.001) (Figure 2). When asked why a patient did not seek formal care as their first treatment of choice, the most commonly cited reasons were thinking that the injury was not serious (N = 78, 43.1%), patient preference (N = 57, 31.5%), and financial reasons (N = 25, 13.8%).

### Patterns and Predictors of Disability

#### Characteristics of Subjects and Disability

**Figure 3:**
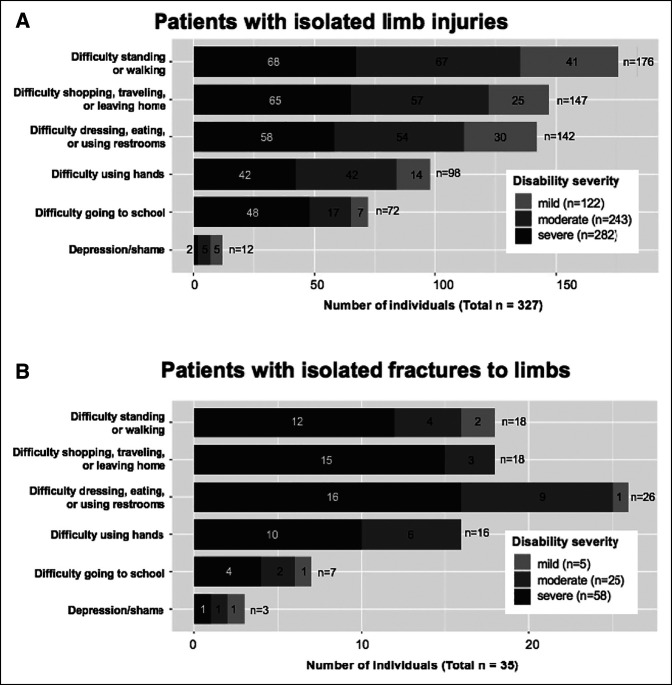
Disability severity reported by individuals with isolated limb injures. **A,** Disability severity reported by individuals with isolated limb injuries. Various areas of disability include (i) having feelings of depression or shame, (ii) difficulty using one’s hands, (iii) difficulty going to school, (iv) difficulty standing or walking, (v) difficulty in leaving home, traveling, or shopping, and (vi) difficulty with dressing, eating, or using the restroom. Of note, the same individual can report multiple areas of disability and were not limited to only one area. **B,** Disability severity reported by individuals with isolated fractures to the extremities.

Of those reporting isolated limb injuries, 76.6% (N = 277) reported having disability for at least 1 day after their injury. Among subjects with limb injuries, 36.2% (N = 131) reported severe disability (unable to perform activities per WHO guidelines^16^) and 29.6% (N = 107) reported moderate disability (required assistance to perform activities^16^). Overall, 49% (N = 176) reported difficulty walking or standing; 41% (N = 147) reported difficulty leaving home, traveling, or shopping; and 39% (N = 142) reported difficulty with activities of daily living including dressing, eating, or going to the bathroom (Figure 3). The disabilities reported led injured subjects to lose a median of 15 days (IQR, 6 to 30) of their primary occupational activity over the 1-year reporting period. When asked about the expected duration of their injuries, 65.2% (N = 234) anticipated or had achieved full recovery; 2.2% (N = 8) anticipated their injury would continue for days; 6.7% (N = 24) anticipated weeks of additional disability; 6.1% (N = 22) anticipated their disability would continue for months; and 1.7% (N = 6) reported disability that their disability was expected to last years or be indefinite.

Nearly all subjects who sustained fractures reported disability after injury (N = 33, 91.7%). In addition, subjects with fractures more commonly reported more severe disabilities compared with patients with other types of limb injuries (68.6% versus 32.7% reporting severe disability in at least one area, *P* < 0.001). Overall, 74% (26) had difficulty performing activities of daily living and over half (N = 18, 51.4%) had difficulty leaving home, traveling, or shopping (Figure 3, B). In addition, individuals with fractures had 30 median days (IQR, 14 to 90) of primary occupation loss because of disability compared with 7 days (IQR, 3 to 30) for those with other types of limb injuries (*P* < 0.001).

Individuals who reported disability reported higher proportions of fractures, sprains, and dislocations (*P* = 0.0003) (Table 1). No notable difference was observed in age, occupation, education level, home location (rural or urban), or mechanism of injury between individuals who reported disability and those who did not.

#### Predictors of Disability

Injury type, mechanism, treatment patterns, and first responder status all were predictive of disability in univariate analysis and were included in multivariate regression building (Supplemental Table 2, http://links.lww.com/JG9/A258). In our stepwise multiple logistic regression model, predictors of developing any type of disability included limb fracture (OR, 5.3; 95% CI, 1.2 to 23.4) and blunt force mechanism (OR, 8.2 in comparison with RTIs; 95% CI, 1.8 to 37.0) (Supplemental Table 3, http://links.lww.com/JG9/A258). Limb fracture (OR, 3.6; 95% CI, 1.0 to 13.1), having a friend as the first responder (OR, 2.1; 95% CI, 1.1 to 4.1), and use of traditional healers as the initial form of care (OR, 2.8; 95% CI, 1.1 to 7.1) were found to predict moderate-to-severe injury (Supplemental Table 3, http://links.lww.com/JG9/A258).

### Economic Effects of Injury

**Figure 4:**
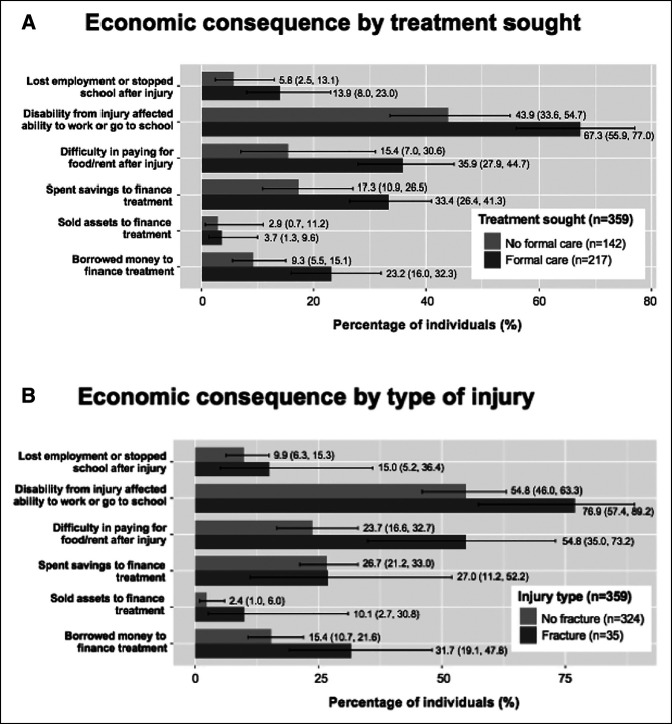
Economic consequences of isolated limb injuries. **A,** Economic consequences of isolated limb injuries by treatment type: Comparison of individuals with isolated limb injuries who sought formal care to those who did not (95% confidence intervals reported). **B,** Economic consequences of isolated limb injuries by injury type: Comparison of individuals with isolated limb injuries who sustained fractures to those who sustained other types of injuries (95% confidence intervals reported).

Over 10% of persons with isolated limb injuries (10.5%; 95% CI, 6.8 to 15.9) lost employment or stopped attending school, and 27.4% (95% CI, 20.4 to 35.7) had difficulty paying for rent or food after injury. There were higher rates of inability to attend school or work among injured individuals who sought formal care in comparison with those who did not seek formal care (67.3%; 95% CI, 55.9 to 77.0 versus 43.9%; 95% CI, 33.6 to 54.7) (Figure 4, A). In addition, compared with those who did not present to formal care, the formal care cohort more frequently financed injury treatment by spending savings (17.3%; 95% CI, 10.9 to 26.5 versus 33.4%; 95% CI, 26.4 to 41.3) or borrowing money (9.3%; 95% CI, 5.5 to 15.1 versus 23.2%; 95% CI, 16.0 to 32.3). The cost of treatment was a median of 7,000 Central African CFA Francs (IQR, 1,500 to 25,000), which is equivalent to approximately 12.0 US Dollars (IQR, 2.6 to 43.2 USD) (Supplemental Table 1, http://links.lww.com/JG9/A258). Individuals who sustained fractures reported higher rates of difficulty in paying for food or rent after injury compared with counterparts with other injury types (54.8%; 95% CI, 35.0 to 73.2 versus 23.7%; 95% CI, 16.6 to 32.7) (Figure 4, B).

## Discussion

We present data from the first large-scale population-level surveillance study in Central Africa reporting specifically on patterns of extremity injury and disability. Because many injured persons in LMICs never interface with formal medical care, our results increase available knowledge about the true epidemiologic distribution and burden of long-term disability resulting from limb injury in this setting. We identified that isolated limb injuries are extremely common and incur high rates of disability. Importantly, individuals who sustained fractures reported higher rates of disability and increased economic hardship and were more likely to seek alternative medical treatments before engaging in formal care.

Our data bolster studies from other LMICs which have found falls and RTI to be the primary mechanisms responsible for limb injuries.^5,7,20,21^ A systemic review of African RTIs estimated that rates more than doubled from 40.7 per 100,000 in the 1990s to 92.9 per 100,000 people, and unfortunately, these rates are projected to increase as industrialization continues to expand.^22^ Country-specific system factors that likely contribute to limb injury in Cameroon include the prevalence of moto-taxis (exposed limbs are at high risk of injury), overcrowding, lack of seat belt utilization in four-wheeled vehicles, poor road infrastructure, minimal training requirements for commercial and private vehicles, and underenforcement of accepted road safety standards.^23,24^ To mitigate physical and economic disability in Cameroon, efforts targeting these deficits should be commensurated to the projected expansion of road utilization. Policy and education interventions that enforce seat belt use, enforce adherence to speed limits, and set minimal safety requirements for motorcycles have markedly improved safety in other regions.^25^ Infrastructure improvements in road quality and public transportation in Cameroon would be expected to further curtail RTI-associated disability rates attributable to limb injuries and potentiate economic development.

As previously described, a key limitation of hospital-based data is the inability to accurately characterize injury epidemiology and therapeutic itinerary in settings where many people do not receive formal care. In this study, we demonstrate that this pattern is particularly prevalent among Cameroonians with isolated limb injuries where only 61% ultimately engage in formal care. Our study found that a higher proportion of persons who sought formal care were unable to return to work or school after their injury, suggesting that Cameroonians may be more likely to seek formal care when their injuries are more severe and likely to result in disability. We also identified high rates of primary or co-management of limb injuries by traditional healers and home remedies. These findings are similar to those of other population studies on LMICs.^4,6,13,26^ Notably, in our study, initial utilization of traditional healers was strongly predictive of developing moderate-to-severe disability after limb injury. Similarly, previous studies in Nigeria found that 69.3% of injured persons preferred traditional bonesetters to formal care,^27^ but that injured patients first treated by a bonesetter had high rates of subsequent malunion (58%) and nonunion (25%).^28^ Although the cross-sectional design of our study limits our ability to know precisely why Cameroonians experience worse disability after traditional medicine, these findings may reflect differences in injury severity, access to rehabilitation services, timeliness of treatment, or disparities in training between traditional healers and formally trained medical personnel. Factors previously identified as contributors to the preference for traditional healers have included mistrust in the formal healthcare system; personal or cultural beliefs; and difficulty accessing formal care because of geographic, language, and economic barriers.^6,26,29^ Given these barriers, potential system targets for decreasing the morbidity attributable to limb injuries may include improving geographic and economic access to formal care. In addition, some LMIC partners have considered or implemented programs that train and partner with traditional medicine providers to improve local outcomes.^30,31^ Training tailored for community bonesetters could potentially increase timely recognition of severe injuries and improve timeliness to evaluation in a formal care setting.^32^

We demonstrated that limb injuries resulted in substantial and often severe disability, with a small but notable subset of individuals anticipating permanent disability. Persons who sustained limb injuries incurred notable economic hardships including lost employment, leaving school, and difficulty affording basic necessities. Comparable with data from other studies,^7,23,29,33^ we found that most individuals who developed disability from limb injuries in Cameroon were between 22 and 50 years, during the most economically productive years of life. As such, the economic burden of limb injury includes both direct treatment costs but perhaps even more markedly considerable loss of future productivity, which can have far-reaching effects on families and regional growth.^7^ Notably, limb fractures were the greatest contributor to disability and economic hardship, resulting in both more severe and longer durations of disability. These findings highlight again the need to target measures to decrease the overall burden of limb injuries and to investigate alternative financing schemes to decrease out-of-pocket expenditures for patients using formal care.

Although interviews were conducted verbally in each family's native language, the study design is subject to limitations of reporting and recall bias. A recall period of 12 months was used to minimize recall inaccuracies and memory decay.^6^ Because all data were self-reported, there may be inaccuracies due to memory decay, under-reporting of sensitive information concerning self-inflicted or intentional injuries, and lack of knowledge on the part of respondents for less severe injuries.^6,34^ Furthermore, the severity of the injury alters the effect of memory decay; previous studies have shown that more severe injuries are subject to less recall inaccuracies.^34^ Our sampling strategy was designed to elicit representative data for the target population, but generalizability to other LMICs, even in sub-Saharan Africa, may be limited. Given the community-based reporting inherent to the study design, we did not include questions on granular clinical data including injury severity, open versus closed fracture, specific diagnostic and treatment modalities, appropriateness of treatment modalities, and complications resulting from these injuries, such as malunion, nonunion, or infection. These data are important to optimize formal treatment of limb injuries and diminish disability in Cameroon but should be investigated in the hospital setting.

## Conclusion

Limb trauma comprises most traumatic injuries sustained in Southwest Cameroon and results in considerable physical and economic disability for young adults in their most productive years of life. Preventive public health measures such as enforcement of road safety laws and improvement of transportation infrastructure are essential to offset injuries resultant from expanding industrialization. Fractures and utilization of traditional rather than formal care services were associated with higher levels of disability after limb injury. Expanding access and acceptability of formal healthcare services and decreasing the economic burden of formal care utilization are essential efforts needed to reduce the disability attributable to these prevalent injuries.
